# Physician’s Right to Refuse Calls

**Published:** 1900-08

**Authors:** 


### Physician's Right to Refuse Calls.

The lay press in Great Britain and in this country has been recently handling the old theme whether a physician has the right to refuse to answer calls. A London coroner's jury, it seems, denounced as inhuman the action of some doctors in refusing to attend a case, and the papers followed its lead. A prominent American journal admits the legal right of the physician to refuse his services, but says that more is expected from him than from the ordinary tradesman, and that we look for some spirit of self-sacrifice from the medical profession. The doctor, according to popular notions, must give his services wherever wanted and at all times, without regard to his own personal comfort or welfare, and his refusal is counted as inhumanity. This is a satisfying sort of self-righteousness, easily assumed, that disposes of other people's rights so freely. A physician has as good a right to refuse to answer a call for his services as has a mechanic or a lawyer, and if he has good reasons, such as fatigue or overwork, no just charge of inhumanity can be made against him. He has also as good a right to refuse to work for nothing as any other man, and it would be better for the profession collectively if he often availed himself of this right. There is no profession that does as much for charity as the medical, and this is true to such an extent that a large section of the public has come to regard what is thus done for them as in the natural order of things and deserving of no acknowledgement. No one wishes to see any moral deterioration or increase of commercialism in the profession, and, indeed, there is no danger of its members as a class repudiating the honorable traditions from the past, but there is no need, on the other hand, that they should acquiesce in the unjust assumptions of pseudo-philanthropic posers, to lay down their rules of conduct and morals for them. That London jury was very probably made up of men who would, in their way of business, do far worse than the doctors whom, in their vicarious conscientiousness, they condemned, without scruple or remorse. As physicians we wish to exercise charity for all, even the offensively self-righteous, but the common, cheap virtue of this kind in the daily press sometimes makes us tired.—*Journal A. M. A.*

